# Editorial: Skin Autoimmunity

**DOI:** 10.3389/fimmu.2021.627565

**Published:** 2021-03-25

**Authors:** Khalaf Kridin, Katja Bieber, Christian D. Sadik, Michael P. Schön, Gang Wang, Karin Loser, Ralf J. Ludwig

**Affiliations:** ^1^ Lübeck Institute of Experimental Dermatology and Center for Research on Inflammation of the Skin, University of Lübeck, Lübeck, Germany; ^2^ Azrieli Faculty of Medicine, Bar-Ilan University, Safed, Israel; ^3^ Department of Dermatology, Allergy, and Venereology and Center for Research on Inflammation of the Skin, University of Lübeck, Lübeck, Germany; ^4^ Department of Dermatology, Venereology, and Allergology, University Medical Center Göttingen, Göttingen, Germany; ^5^ Lower Saxony Institute of Occupational Dermatology, University Medical Center Göttingen, Göttingen, Germany; ^6^ Department of Dermatology, Xijing Hospital, Fourth Military Medical University, Xi’an, China; ^7^ Institute of Immunology, University of Oldenburg, Oldenburg, Germany

**Keywords:** skin, autoimmunity, pemphigus, pemphigoid, psoriasis, alopecia aerata (AA)

## The Spectrum of Skin Autoimmune Diseases

According to the revised Witebsky’s criteria by Rose and Bona, a disease is considered of autoimmune origin if (i) it can be transferred by pathogenic T cells or autoantibodies, (ii) it can be induced in experimental animals, or if (iii) autoimmunity is suggested by circumstantial evidence from clinical clues (1). Rather new aspects are the iatrogenic induction of autoimmune side effects or the emergence of cutaneous side effects by new immunomodulating therapies (2, 3). According to the “classical definition”, skin autoimmune diseases include pemphigus and pemphigoid diseases (4, 5). In pemphigus and pemphigoid, autoantibodies bind to specific structural proteins of the skin and either directly or indirectly (through activation of the immune system) induce skin pathology (6). Within this Research Topic, several review articles provide an excellent overview of several pemphigus and pemphigoid diseases. More specifically, paraneoplastic pemphigus, bullous pemphigoid, anti-p200 pemphigoid and lichen planus pemphigoides are reviewed in detail. Within this editorial, we aim to provide an overview of the 68 articles of the Research Topic. Each article of the Research Topic can be directly assessed by the kinks provided in the blue font. To guide the reading, we have classified the articles into the following subheadings:

- “Emerging” autoimmune diseases
- Novel insights into the pathogenesis of skin autoimmune diseases
- New diagnostic approaches in skin autoimmune diseases
- Comorbidity in skin autoimmune diseases
- Epidemiology of skin autoimmune diseases
- Novel treatment targets and therapeutic approaches for skin autoimmune diseases
- Characterization of patient biomaterials and model systems of skin autoimmune diseases

## “Emerging” Autoimmune Diseases

In contrast to these more “classical” autoimmune diseases, fulfilling the revised Witebsky’s criteria, there is an increasing evidence for a role of autoreactive T- and/or B-cells in chronic inflammatory skin diseases that have not been considered autoimmune so far (7). As reviewed by Boehncke and Brembilla autoreactive T cells are present in several chronic skin inflammatory diseases that are (so far) not considered to be caused by an aberrant immune response to self-antigens. Among others, autoreactive T cells have been identified in psoriasis and atopic dermatitis (8–10). Overall, these are very intriguing findings, but before fully considering autoimmunity as a significant contributing factor to psoriasis or atopic dermatitis, these findings require functional validation. In addition to T cell-mediated autoimmunity in “non-autoimmune” chronic inflammatory skin diseases, presence of autoantibodies has been described in chronic spontaneous urticaria (CSU), as well as in morphea. Yet, again, these findings await functional validation *in vivo*. The induction of systemic sclerosis, which shares pathogenic features of morphea, in mice immunized with type V collagen (11), however, provides strong evidence for a pathogenic contribution of autoantibodies in morphea and/or systemic sclerosis. In light of the increasing importance of pathogenetic networks of innate and adaptive immune responses (12, 13), this and other observations are highlighted in the review by Schön, where in psoriasis and other autoimmune or autoinflammatory diseases, and their relation to disease pathogenies is highlighted.

## Novel Insights Into the Pathogenesis of Skin Autoimmune Diseases

A complex interaction of genetics and environmental factors is one of the key underlying pathogenic mechanisms in skin autoimmune diseases. The high number of submissions reporting on genetic associations in autoimmune skin diseases underscores this. The genetics and transcriptomics in pemphigus and pemphigoid are reviewed by Olbrich et al. Targeted genetic analysis identified novel gene polymorphisms in endemic pemphigus foliaceus, namely within cell death pathways and the soluble CR1. While most genetic studies focus on the nuclear genome, few address the impact of the mitochondrial genome on complex phenotypes. This highly interesting topic has been addressed by Russlies et al., who report on polymorphisms in the mitochondrial genome that are associated with bullous pemphigoid.

In addition to genetics, several articles also specifically address certain cell types in skin autoimmune diseases. Cao et al. allude to the role of regulatory immune cells in pemphigus and pemphigoid. Here, with a focus on pemphigus and pemphigoid, they review the impact of different types of regulatory T and B cells. Rauschenberger et al. present data on the crosstalk between skin infiltrating T cells and keratinocytes. Costa et al. focus on the contribution of mononuclear phagocyte activation in the context of psoriasis. Based on the determination of molecular markers of monocyte/phagocyte activation in inflamed skin and the serum of patients, they conclude that mononuclear phagocytes are activated in psoriasis and thus may contribute to disease pathogenesis. Interestingly, similar findings were made in patients with bullous pemphigoid by Riani et al. Neubert et al. also focus on the contribution of innate immune cells in the pathogenesis of chronic skin inflammation: Neutrophil extracellular traps (NETs) may be formed after neutrophil activation. NETs are important for host defense, but may also contribute to the pathogenesis of several chronic inflammatory (skin) diseases, such as rheumatoid arthritis, psoriasis, or systemic lupus erythematosus (SLE). Interestingly, some of these diseases may be triggered or aggravated by light exposure. Based on these findings, the authors investigated the impact of UVA on NET formation.

Another set of articles within the Research Topic focus on the role of cytokines in the pathogenesis of chronic skin inflammation: Buhl and Wenzel highlight the importance of the different isoforms IL-36, especially in psoriasis. The contribution of IL-36 in (pustular) psoriasis is underscored by the induction of pustular psoriasis in carriers of a missense mutation of the IL-36Ra. Le Jan et al. identified inflammasome activation in patients with bullous pemphigoid. Increased expression of IL-1β was induced by IL-17/-23 and led to an increased release of proteases from macrophages. Increased IL-1β expression was, however, predominantly found in patients with erythema and urticarial lesions. Thus, determination of IL-1β concentrations may allow to stratify patients in personalized treatment approaches.

Further articles focused on the pathogenesis of chronic inflammatory skin diseases: Liu et al. report on the effects of TIPE2, a members of the tumor necrosis factor-α induced protein-8 family, in mouse models of psoriasis and autoimmune uveitis. Interestingly, in TIPE2-deficient mice opposing effects were observed: While TIPE2-deficiency alleviated clinical disease manifestation in Aldara-induced psoriasiform dermatitis, development of experimental autoimmune uveitis was exacerbated. In his article, Arneth performed a systematic literature search on SLE and summarizes the evidence that SLE is a disease caused by a disorder of DNA degradation and elimination. While the review by Günther covers a similar topic, she points toward the type I interferon signature in lupus as well as environmental triggers of the disease. Two articles provide insights into the pathogenesis of pemphigoid diseases: Hiroyasu et al. summarize the contribution of proteases to pemphigoid disease pathogenesis. Jegodzinski et al. investigated the impact of the G protein-coupled receptor 15 (GPR15) on experimental pemphigoid disease. Of note, they identified GPR15 as one (of the few) anti-inflammatory molecule in pemphigoid diseases.

## New Diagnostic Approaches in Skin Autoimmune Diseases

The molecular-based diagnosis of pemphigus and pemphigoid, with more detailed insights into mucous membrane pemphigoid, is also reviewed within this Research Topic. In addition to serology, novel non-invasive methods have been developed for the diagnosis of a variety of chronic inflammatory and autoimmune skin diseases. Among these are imaging systems such as optical coherence tomography, as well as non-invasive sampling methods such as plucked hairs and tape-stripping from skin. All of these are used for the diagnosis of disease. Recently, diagnostic developments have also focused on the development biomarkers to predict disease outcome and/or response to treatment (14). As an example, Nesmond et al. identified IL17RA and IL17RC expression on monocytes to be associated with disease activity in bullous pemphigoid.

## Comorbidity in Skin Autoimmune Diseases

With the availability of (relatively) effective treatments for chronic skin inflammation (15–17), comorbid diseases, mostly metabolic and cardiovascular, now significantly contributes to the morbidity of patients with skin autoimmune diseases. For example, an increased prevalence was noted in psoriasis inpatients well over 50 years ago (18). Several decades later, an increased prevalence of coronary artery calcification and myocardial infarction in patients was demonstrated in imaging and epidemiologic studies (19, 20). Within this Research Topic Cugno et al. review the evidence for the prothrombotic state in CSU and bullous pemphigoid. Of note, inflammation and the prothrombotic state may form a vicious circle, whereby each pathway sustains and promotes the activation of the other. This has been compellingly reviewed in this Research Topic by Liu et al.

In addition to the metabolic and cardiovascular comorbidity of patients with autoimmune skin diseases, a high prevalence of neurologic disease has been noted in bullous pemphigoid patients (21, 22). Mechanistically, the expression of one of the major BP autoantigens, BP180 in the brain has been linked to this clinical association (23). Herein, Wang et al. demonstrate that in patients with stroke, autoantibodies against BP180 were more prevalent compared to healthy controls. This suggests that autoantibodies against BP180 are relatively common after stroke. Consequently, clinical manifestation of bullous pemphigoid could emerge after stroke. However, epitope spreading may be needed because in multiple sclerosis and Alzheimer’s disease, which are also associated with bullous pemphigoid, recognize different epitopes of BP180 than those bound by bullous pemphigoid patients (23).

## Epidemiology of Skin Autoimmune Diseases

During the last years, evidence has accumulated that bullous pemphigoid may be triggered by certain medications. More specifically, use of checkpoint inhibitors and dipeptidyl peptidase-4 inhibitors (DPP4i) is significantly associated with bullous pemphigoid (24). Within the Research Topic, the association of bullous pemphigoid with DPP4i use is reviewed. This topic is also addressed in a cross-sectional study comparing the prevalence of bullous pemphigoid autoantibodies in patients with type II diabetes treated with or without DPP4i. Here, the authors demonstrated that BP180NC16A autoantibodies were more prevalent in those diabetics treated with DPP4i. Analysis of different DPP4i showed that the increased prevalence of anti-BP180-NC16A was observed only for some, but not all DPP4i. Regarding checkpoint inhibitor-induced bullous pemphigoid, the Research Topic includes one interesting case report: A patient suffering from metastatic melanoma was treated with nivolumab, leading to a partial remission. After approximately 5 months, anti- thyroid peroxidase autoantibodies and hypothyroidism developed. At the same time, bullous pemphigoid was diagnosed. Relating to bullous pemphigoid, intriguingly only LAD-1 autoantibodies were detected. This case reports is in line with previous cases that, however, so far had only reported on Japanese patients presenting with LAD-1-only bullous pemphigoid. Another important, so far neglected field of research in bullous pemphigoid, are insights into demographics and clinical data from the USA. This has been addressed by Lee et al. in this article collection. They demonstrate female predominance in both bullous pemphigoid and mucous membrane pemphigoid. Furthermore, a large number of bullous pemphigoid and mucous membrane pemphigoid presented with other autoimmune diseases; most commonly thyroid disease. Another important aspect of this study is the notion of severe limitations of daily activities imposed by these diseases in the patients. A significant decrease in the quality of life and an inverse correlation with disease activity is also reported for pemphigus patients. One article addresses the impact of airborne pollution with dermatomyositis, underscoring the importance of the environment modulating the susceptibility to develop autoimmune disease (25, 26).

## Novel Treatment Targets and Therapeutic Approaches for Skin Autoimmune Diseases

Despite recent advances in the treatment of autoimmune skin diseases there is still a high medical need to develop new treatments that are more effective and safer, as well as induce long-term remissions. This need is for example reflected by pemphigus. Here, the combination of systemic corticosteroids with the anti-CD20 antibody rituximab is far more effective and induces dramatically less adverse events compared to treatment with systemic corticosteroids (albeit at a higher dose) alone (27). Yet, the time to remission still requires 180 days (27) and, as documented by Mohamad et al. in this Research Topic, relapses are frequent, even during tapering of the corticosteroid dose. One possible approach to meet this medical need may be the use of (modified) preparations of high dose intravenous immunoglobulins (IVIG) as highlighted by Hoffman and Enk.

In bullous pemphigoid several polymorphisms in genes encoding for cytokines have been described (28), some of which have been functionally validated in pemphigoid disease animal models (29–31). Herein, polymorphisms within IL8 were observed when contrasting patients and controls. The *IL8* polymorphisms also translated into an increased mRNA expression of the cytokine. Over the last years evidence has accumulated that points toward the presence of IgE autoantibodies on pemphigoid diseases (32, 33). The pathogenic relevance of IgE in pemphigoid has been demonstrated in experimental bullous pemphigoid, where the disease was induced by injecting anti-COL17 IgE into mice humanized for (parts of) COL17 and the IgE receptor (34). In clinical settings, treatment of bullous pemphigoid patients with the anti-IgE antibody omalizumab has been reported to have beneficial effects. Within the Research Topic, Jafari et al. also report on the successful treatment outcome in 2 bullous pemphigoid patients after omalizumab. They furthermore provide insights into molecular changes that are associated with clinical improvement of the disease. Of note, down-regulation of FcϵRI, as well as IgE in lesional skin and on circulating basophils were observed to be associated with clinical improvement.

In addition to cytokines and potentially IgE autoantibodies, the activation of the complement system is of key importance to mediate skin inflammation and subepidermal blistering in pemphigoid diseases (35). This has already led to clinical trials investigating the safety and efficacy of complement-targeting biologics in pemphigoid diseases (36, 37). This topic is reviewed in detail in the Research Topic. In addition, Zheng et al. here provide compelling evidence that the central hub of complement activation, the C5a/C5aR1 is also critical for the pathogenesis of psoriasis. In the Aldara-induced psoriasiform dermatitis model, as well as in the IL-23-induced model of psoriasis genetic or pharmacological inhibition of the C5aR1 significantly ameliorated the onset of clinical disease. Mechanistically, this seems to be linked to the migration and differentiation of plasmacytoid dendritic cells.

Lichen planus is a chronic relapsing inflammatory disorder of the skin and mucous membranes. The disease is characterized by band-like T cell infiltrates along the dermal-epidermal junction and apoptosis of basal keratinocytes (38). More recently, compelling data has emerged that documents the presence of desmogelin 3- and COL17-specific, autoreactive T cells in lichen planus. Characterization of the T cell response in lichen planus showed a polarization toward Th1 and Th17 cells. In addition, IL-17-producing cells were found to be present in the skin of lichen planus patients (39). Herein, Solimani et al. follow-up on these observations and report the treatment outcomes of 3 lichen planus patients treated with anti-IL17 (secukinumab). In all 3 patients, a favorable clinical outcome was observed, that was accompanied by a reduced dermal T cell infiltrate. In addition to targeting T cell-derived cytokines, modulation of T cell metabolism is an alternative, but promising approach to treat chronic skin inflammation.

One major focus of the articles submitted to the Research Topic was inhibition of Janus kinases (JAK). Based on elaborate pre-clinical work that encompassed genetic studies, transcriptomics as well as use of pre-clinical model systems of chronic skin inflammatory diseases the JAK pathway was identified as a potential therapeutic target for several chronic inflammatory skin diseases (40, 41). In the review by Szilveszter et al., the various tyrosine kinase signaling pathways and their role in autoimmune and inflammatory skin diseases is presented in detail. The focus is on ongoing preclinical trials and clinical studies using small-molecule tyrosine kinase inhibitors in chronic inflammatory diseases. The reviews by Solimani et al. and by Howell et al. focus on the immunological basis and current stage of development of JAK inhibitors in chronic inflammatory indications in dermatology. However, as proposed by Ehrchen et al., insights into the mode of action of “old” drugs, such as glucocorticoids may lead to the identification of novel therapeutic targets.

As highlighted by Zeidler et al., pruritus is a highly prevalent in chronic autoimmune diseases, and often is one of the key factors contributing to patient morbidity (42). Of note, and highlighted herein, IL-31 may be one of the key drivers of pruritus. The potential importance of targeting IL-31 to alleviate itch and certain chronic inflammatory skin diseases is highlighted by several clinical trials that have and are evaluating the safety and efficacy of anti-IL-31 treatment (43, 44).

In addition to the above articles, CCR2-A in chronic cutaneous lupus erythematosus, neutrophil extracellular traps in Schnitzler’s Syndrome and the skin barrier in pemphigus and pemphigoid have emerged as potential targets and treatment approaches.

## Characterization of Patient Biomaterials and Model Systems of Skin Autoimmune Diseases

Clinical and molecular characterization of patients and biomaterials, as well as model systems are an important pilar to understand pathogenetic pathways and identify novel therapeutic targets. As an example for an in-depth characterization of patient samples is the description of a protocol that allows the identification of autoreactive B cell subpopulations in the peripheral blood in patients with pemphigus. Another use of patient-derived biomaterial is its’ functional evaluation, exemplified by the Walter et al., who contrasted the signaling in keratinocytes after binding of either anti-Dsg 1 or anti-Dsg 3 IgG binding. This work demonstrates that pathogenic pathways in pemphigus differ depending on the autoantibodies. Therefore, exact determination of the autoantibody profile in pemphigus patients by the respective serological test systems (45, 46) is the key to personalized treatment approaches in pemphigus.

Several articles of this Research Topic described novel animal models or provided in-depth reviews: In most pemphigus patients, autoantibodies against Dsg 1 and/or 3 are present (5), while non-Dsg autoantibodies are found in a minority of patients (47). This may be one of the main reasons, why most pemphigus animal models mirror the immune response against Dsg 1 and/or 3 (48). Only recently, Lotti et al. described a pemphigus mouse model that is based on the transfer of lymphocytes from mice immunized with desmocollin into immunodeficient mice. In addition to this novel pemphigus mouse model, new findings in scurfy mice are described, namely the development of mixed connective tissue disease, and mouse models of Sjögren’s Syndrome are reviewed. Demeyer et al. report on the development of atopic-like dermatitis in ageing MALT1-deficient mice. Given further validation, this new model of atopic dermatitis may be useful to decipher pathogenic pathways in atopic dermatitis.

## Concluding Remarks

Overall, the field of skin autoimmunity has become a major focus, both in research and clinical practice. This is also reflected by the 68 submissions to the Research Topic that provides state-of-the art reviews, as well as novel insights into skin autoimmune diseases. Overall, each novel insight into the pathogenesis of these severe disease provides clues to better treat and diagnose affected individuals.

## Author Contributions

All authors contributed to the article and approved the submitted version.

## Funding

This work supported the Excellence Cluster “Precision Medicine in Chronic Inflammation” (EXC 2167) from the Deutsche Forschungsgemeinschaft. KK received support from the Alexander von Humboldt Foundation (Humboldt Research Fellowship for Postdoctoral Researchers).

## Conflict of Interest

MS has received honoraria from AbbVie, Biogen, Almirall, Leo, Novartis, UCB, Janssen, Leo. RL has received honoraria and/or research grants from the following companies: Admirx, Almirall, Amryth, ArgenX, Biotest, Biogen, Euroimmun, Incyte, Immungenetics, Lilly, Novartis, UCB Pharma, Topadur, True North Therapeutics and Tx Cell.

The remaining authors declare that the research was conducted in the absence of any commercial or financial relationships that could be construed as a potential conflict of interest.
